# Molecular basis of canalization in an ascidian species complex adapted to different thermal conditions

**DOI:** 10.1038/srep16717

**Published:** 2015-11-18

**Authors:** Atsuko Sato, Takeshi Kawashima, Manabu Fujie, Samantha Hughes, Noriyuki Satoh, Sebastian M. Shimeld

**Affiliations:** 1 Department of Zoology, University of Oxford, South Parks Road, Oxford OX1 3PS, United Kingdom; 2 Marine Genomics Unit, Okinawa Institute of Science and Technology Graduate University, Onna, Okinawa, 904-0495, Japan; 3 Marine Biological Association of the UK, The Laboratory, Citadel Hill, Plymouth, PL1 2PB, United Kingdom; 4 DNA Sequencing Section, Okinawa Institute of Science and Technology Graduate University, Onna, Okinawa, 904-0495, Japan

## Abstract

Canalization is a result of intrinsic developmental buffering that ensures phenotypic robustness under genetic variation and environmental perturbation. As a consequence, animal phenotypes are remarkably consistent within a species under a wide range of conditions, a property that seems contradictory to evolutionary change. Study of laboratory model species has uncovered several possible canalization mechanisms, however, we still do not understand how the level of buffering is controlled in natural populations. We exploit wild populations of the marine chordate *Ciona intestinalis* to show that levels of buffering are maternally inherited. Comparative transcriptomics show expression levels of genes encoding canonical chaperones such as Hsp70 and Hsp90 do not correlate with buffering. However the expression of genes encoding endoplasmic reticulum (ER) chaperones does correlate. We also show that ER chaperone genes are widely conserved amongst animals. Contrary to previous beliefs that expression level of Heat Shock Proteins (HSPs) can be used as a measurement of buffering levels, we propose that ER associated chaperones comprise a cellular basis for canalization. ER chaperones have been neglected by the fields of development, evolution and ecology, but their study will enhance understanding of both our evolutionary past and the impact of global environmental change.

Canalization is an important part of our understanding of development and evolution^1^,^2^. It describes the ability of individuals in a population to produce essentially the same phenotype despite genetic differences and variation in environmental conditions. As such, canalization can be considered a consequence of the robustness of the mechanism turning genotype into phenotype. Phenotypic plasticities, in which different canalized pathways can be adopted by the same genotype depending on environmental conditions, are more complex examples of this. There are also examples where, in a population of differently canalized forms, if a single canalized form is selected for many generations then that form becomes fixed^3^,^4^.

At the cellular and organismal levels canalization may be explained by buffering mechanisms that maintain developmental robustness in the face of genetic and environmental perturbation. Describing the molecular nature of such mechanisms has begun only recently, initiated by the discovery that Hsp90 can act as a capacitor of morphological variation^5^. Since then, a number of candidate mechanisms have been proposed. These include microRNAs, which may buffer variation in gene expression level^6^,^7^ as well as buffering genomic variation^7^, gene duplication and dosage^7^,^8^, a role for low affinity transcription factor binding sites^9^, and genotype networks^10^. Such studies can be interpreted as support for the hypothesis that buffers limit the appearance of genetic variation in the phenotype under normal conditions, but that environmental stress may titrate buffering and allow genetic variation to affect the phenotype and fall under selection^1^,^5^. Although these studies have advanced our understanding of the mechanisms of canalization, they have been done in model organisms that have been cultured in stable laboratory conditions over a long period of time.

Whilst mechanistic studies on canalization in wild populations are lacking, observational studies of stress tolerance are common. Amongst the possibilities listed above, Hsp90 has been by far the most studied in this context. Even between very closely related species there can be a large difference in environmental stress susceptibility and associated difference in species distributions. Studies have shown differences in expression levels of the gene encoding Hsp90 between closely related species^11^. However, it has not been established whether their expression level can be used as a measure of developmental robustness, or whether this is the only chaperone involved.

There are numerous chaperones encoded in animal genomes, including Hsp90s, Hsp60s, Hsp70s (DnaK), Hsp40s (DnaJ) and small heat shock proteins, very few of which have been investigated in non-model organisms. The DnaJ group is the largest and the most diverse, and encode a DnaJ domain that interacts directly with the Hsp70 ATPase^12^. As such, DnaJs can be viewed as regulators of the canonical protein folding activity of the Hsp70^13^.

The level of buffering can be determined experimentally by comparing the reactions of groups of individuals to an environmental stress^1^. To investigate the questions above and identify the underlying mechanisms, a system is required which combines molecular and embryological experimental tractability with natural populations that differ in buffering capacity. The tunicate *Ciona intestinalis* is one such system. Tunicates are exclusively marine and the closest invertebrate relatives of vertebrates, with *C. intestinalis* an established model system for developmental biology and genomics^14^. It has planktonic embryos and larvae, is highly invasive, and commonly fouls underwater structures^15^. Most significantly for this study, *C. intestinalis* consists of two sibling species, types A and B, with different distributions^16^,^17^,^18^,^19^,^20^,^21^,^22^,^23^,^24^. In Europe, type A is found on the Mediterranean/Atlantic Coast of Southern Europe, where seawater temperature can rise to 28 °C, whereas type B is limited to the Northern European coast where temperature reaches a maximum of 17 °C (http://www.westernchannelobservatory.org.uk/l4_buoy_history.php). A small region of overlap has been identified in the English Channel^19^,^25^, where the two species can be found side-by-side. Previous studies have indicated that hybrids, though rare in nature, are viable in the laboratory^18^,^26^, providing a model system in which maternal and zygotic effects can be distinguished.

Using *C. intestinalis* from a common-garden zone of species overlap in the English Channel, we show that buffering of early development differs between type A and type B. Analysis of hybrids shows buffering is maternally inherited. Transcriptomics and qPCR data show that the expression levels of the genes encoding *dnajc3* and *dnajc10*, but not *hsp83* nor *hsp70*, are also maternally inherited, and correlate with the level of buffering. We further show that the genes encoding *dnajc3* and *dnajc10* are evolutionarily ancient, with orthologues found in early-diverging animal lineages and in the unicellular organisms most closely related to animals. We hence suggest that the deployment of ER-associated DnaJ proteins reflects a conserved mechanism of buffering that is likely to be important in canalizing animal development.

## Results

### Buffering differs between type A and B *C. intestinalis* and is maternally inherited

**Figure 1:**
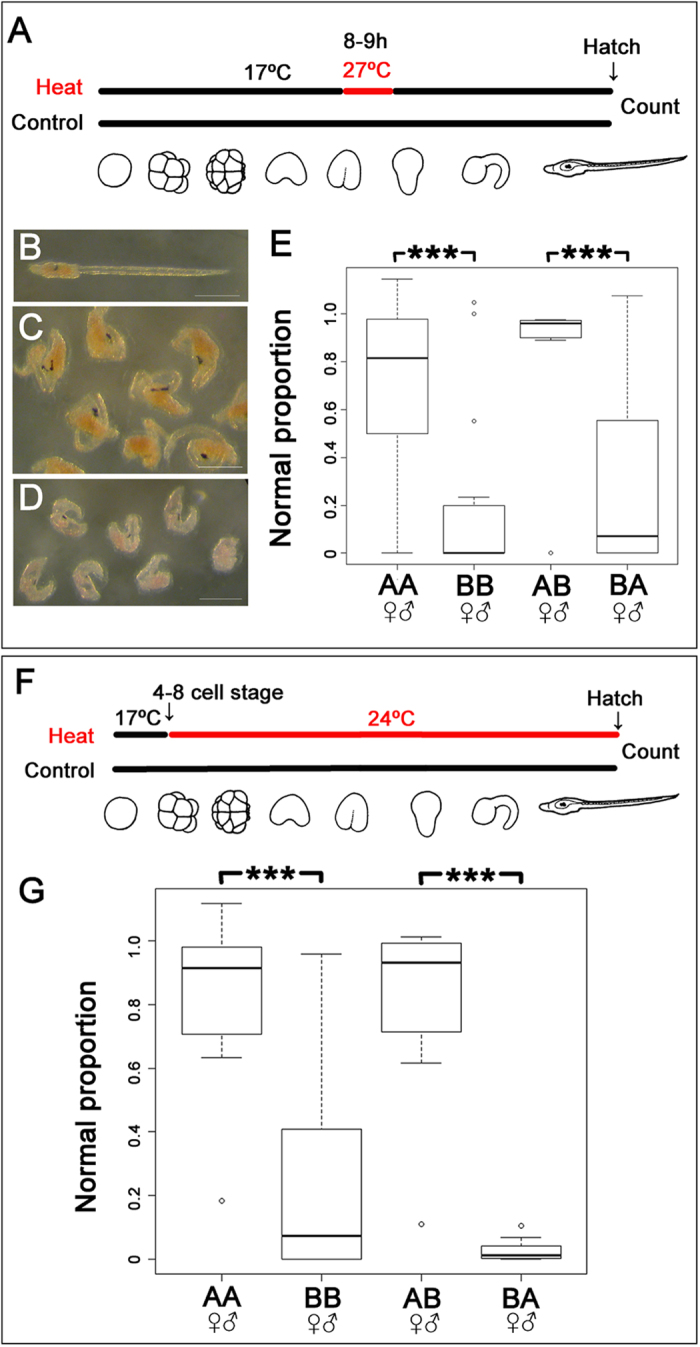
Impact of thermal stress on the development of *Ciona intestinalis* types (A,B). (**A–E**) Impact of short-term exposure to heat shock. Control embryos were cultured at 17 °C. Others were treated at 27 °C for 1 h at the neurula stage, the minimum condition to observe the differences in buffering of thermal stress between types A and B (Supplementary Fig. S1), before returning to 17 °C as schematically illustrated in (**A**). Outcomes vary from normal development (**B**) to truncation of the tail (**C**) to more severe effects on development resulting in a failure to hatch (**D**). Scale bar: 100 μm. (**E**) Comparison of the normal proportion of development (the proportion of normal development in treated embryos relative to the proportion in controls) after heat shock in different genotypes. (**F**,**G**) Impact of long-term exposure at moderately increased temperature. Early cleavage embryos (4–8 cell stage) were reared at 24 °C and control embryos at 17 °C until hatching as shown schematically in (**F**). (**G**) Comparisons of normal proportion after heat shock in different genotypes. The bottom and top lines of the boxes indicate 25^th^ and 75^th^ percentiles, and the horizontal lines how median of each data. The vertical lines show either the maximum value or 1.5 times of the interquartile range of the data, whichever the smaller. In both types of treatment, ANOVA rejected the null hypothesis that the impact of thermal stress does not depend on the genotypes by ***P < 10^−15^. AA, type A conspecific cross; BB, type B conspecific cross; AB, hybrids using type A egg and type B sperm; BA, hybrids using type B eggs and type A sperm.

Seawater temperature can have a significant impact on biological invasions^27^. The observation that the ranges of types A and B *C. intestinalis* are approximately separated by latitude led us to hypothesize that they may be adapted to different seawater temperature ranges. We reasoned that one way this would manifest is that developing embryos would differ in their capacity to buffer temperature stress. To minimize environmental variation we collected *C. intestinalis* types A and B that grow side-by-side in the small region of range overlap in the English Channel. We then conducted *in vitro* fertilizations, cultured batches of embryos from defined parental crosses in the laboratory under controlled conditions, and developed experimental heat shock regimes. We first identified experimental heat shock conditions that differently affected types A and B. A short, high, heat shock of developing embryos (1 h at 27 °C; Fig. 1A) caused a variety of developmental phenotypes to appear, ranging from no observable effect, to abnormalities including truncation of the trunk and tail, deformation of brain and notochord, to complete failure of hatching (Fig. 1B–D). To statistically compare the impact of this treatment on batches of embryos from type A and type B parents, we established a metric of successful development, and coined a term ‘normal proportion’: the frequency of normal larvae in the heat treated experiment divided by frequency of normal larvae in control experiment. Comparison of ‘normal proportion’ between type A and type B larvae after this short heat shock showed type B larvae had a significantly lower normal proportion than type A larvae. This indicates type A embryos have a greater capacity to buffer temperature (Fig. 1E; *P* < 10^−15^). We also tested the impact of a different heat shock regime, in which embryos were exposed to moderately increased temperature for a longer time (24 °C, overnight). This produced a similar range of developmental defects, which were also present at a significantly lower frequency in type A than in type B (*P* < 10^−15^, Fig. 1F,G). These data suggest that the embryos of the two species have different capacities of buffering and that this reflects their ranges, with type A, better able to buffer temperature-induced developmental defects.

The differing ability of types A and B embryos to buffer temperature might reflect differences in zygotic gene transcription. It may also reflect a maternal effect: *C. intestinalis* larvae are non-feeding and have to survive embryonic development, planktonic larval dispersal and metamorphosis on the resources supplied by the mother. Study of hybrids^23^,^26^ can disentangle maternal and zygotic effects, and as hermaphrodites *C. intestinalis* are well suited to this since reciprocal crosses can be generated. We therefore crossed type A and B gametes and subjected hybrid embryos to the same heat shock regimes as described above. For embryos exposed to a short, high heat shock we observed significant maternal inheritance of the level of buffering (*P* < 10^−15^; Fig. 1E), but no effect of the paternal genotype. Similarly, for crosses exposed to the longer, lower heat treatment, buffering ability was also maternally inherited (*P* < 10^−15^; Fig. 1G).

### Molecular correlates of maternally inherited buffering capacity

To identify candidate molecules that are altering buffering levels, we generated transcriptome data for types A and B embryos after heat shock at 27 °C for 1 h. By mapping reads and comparing RPKM values, we found over 10,000 genes, many with unknown functions, which differed in RPKM levels by 0.7 fold or more between types A and B (Supplementary Table S1). Since these transcriptome data are not quantitative, and because there were too many that differed in RPKM to analyze experimentally, we decided to focus on genes encoding chaperone molecules, since these have been the major target of studies investigating levels of buffering under environmental stress in other systems^5^,^11^. From mapped reads we extracted those mapping to 56 such candidate genes, including all the annotated chaperones in the *C. intestinalis* genome^28^, and a variety of other genes implicated in heat shock response in other species (Supplementary Table S2). Transcriptome read ratios between types A and B showed that 28 of these genes had lower read numbers in type B than in type A (Supplementary Table S2).

To statistically confirm this potential interspecific variation^29^, we measured expression levels by qPCR in both control and heat shocked embryos from multiple crosses of both genotypes (Supplementary Figs S1–10). qPCR data showed that those genes with an RPKM ratio between type B and type A of less than 0.7 had significantly lower expression in type B compared to type A (Supplementary Table S3). According to this guide, we prioritized 11 chaperone genes from the list and conducted qPCR analysis. This showed a significant difference in expression between types A and B for 5 genes (*dnajc3, dnajc10, hsp83*, *trap1* and *tcp1theta*) but not for a number of others (*dnajb9*, *dnajc5*, *hsp60, hsp70*, *hspa8*, and *hspa9b*: Supplementary Table S3).

**Figure 2:**
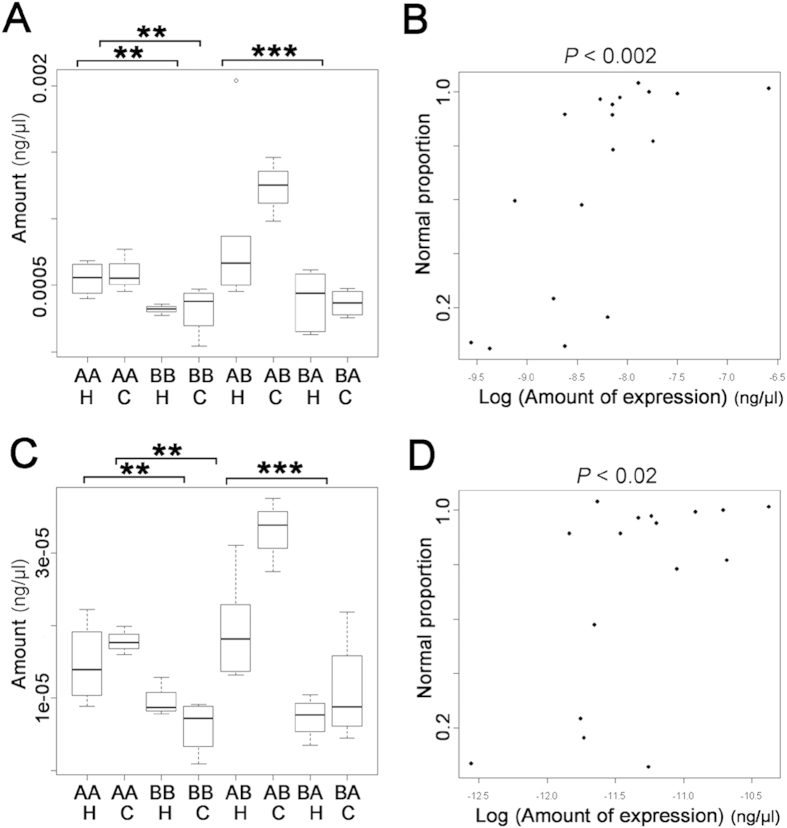
Expression levels of *dnajc3* and *dnajc10* in crosses of different genotype. (**A**) Quantification of *dnajc3* expression levels by qPCR using ‘primer set 2’ (Supplementary Table S4) in control (**C**) or heat shocked embryos (H: 27 °C for 1 h at neurula stage as in Fig. 1A). (**B**) Correlation between the amount of *dnajc3* transcript and normal proportion of development after heat shock in individual crosses. Analysis of co-variance (ANCOVA) rejected the null hypothesis and supported linear correlation of the amount of *dnajc3* transcripts and normal proportion (*P* < 0.002). (**C**) Quantification of *dnajc10* expression levels by qPCR using ‘primer set 1’ (Supplementary Table S4). Note that reciprocal hybridization (AB/BA) showed this was linked to the maternal genotype (***P < 0.0001). (**D**) Correlation between the amount of *dnajc10* transcript and normal proportion of development after heat shock in individual crosses. Analysis of co-variance (ANCOVA) rejected the null hypothesis and supported linear correlation of the amount of *dnajc10* and ‘normal proportion’ (*P* < 0.02). A similar result was obtained from the ‘primer set 2’ (see also Supplementary Figs S1 and S2). Note that heat shock did not affect the expression of both genes, but expression of type A (AA) was significantly higher than in type B (BB) in both genes (**P < 0.001). Reciprocal hybridization (AB/BA) also showed this was linked to the maternal genotype (***P < 0.0001).

As described above, analysis of hybrid embryos showed that buffering levels were maternally inherited, leading us to predict that expression of genes conferring buffering levels would also be maternally inherited. We therefore tested maternal inheritance by examining transcript level in hybrid embryos. We did not find maternal inheritance of transcript levels for many of the genes commonly regarded as encoding buffers of thermal stress, such as *hsp70*, *hsp83* (which encodes Hsp90) and *hsp60*^5^,^11^,^30^ (Supplementary Figs S1–10). Instead, we found levels of *dnajc3*, *dnajc10* (also known as *ERdj5*), *dnajb9, trap1* and *tcp1theta* were maternally inherited (Supplementary Table S3; Fig. 2A,C). Furthermore, we tested for correlation between the level of buffering (as determined by normal proportion) and the expression level of each gene, considering all genotypes and both control and heat shock conditions. The level of buffering was significantly correlated to the expression level of *dnajc3* and expression level of *dnajc10*, but not to that of other genes (Fig. 2B,D).

### The *dnajc3*, *dnajc10*, and *dnajb9* genes are evolutionarily ancient

**Figure 3:**
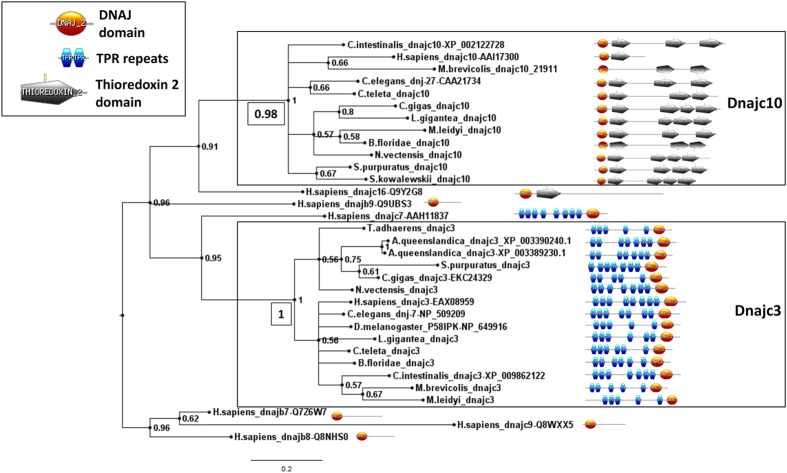
Molecular phylogenetic analysis of prospective *dnajc3* and *dnajc10* sequences, plus a selection of outgroup DnaJ domain sequences. The tree shown was produced by Bayesian analysis and values adjacent to nodes are posterior probabilities. Additional values in boxed text next to the nodes uniting the *dnajc3* and *dnajc10* clades are derived from Maximum Likelihood analysis, and the full tree from which these have been extracted can be seen in Supplementary Fig. S11. The domain structure of each predicted protein is shown on the right. Note the carboxy and amino terminal placement of the DnaJ domain in Dnajc3 and Dnajc10 proteins, respectively. Variable numbers of predicted TPR repeats and Thioredoxin 2 domains may reflect the stringency criteria used in domain prediction and should not be taken as necessarily biologically informative. Diagrams are not to scale. Species included in the figure are *Drosophila melanogaster*, *Caenorhabditis elegans*, *Homo sapiens*, *Ciona intestinalis*, *Branchiostoma floridae* (amphioxus), *Strongylocentrotus purpuratus* (sea urchin), *Saccoglossus kowalevskii* (acorn worm), *Lottia gigantea* (mollusc), *Crassostrea gigas* (mollusc), *Capitella teleta* (annelid), *Nematostella vectensis* (anemone), *Mnemiopsis leidyi* (ctenophore), *Amphimedon queenslandica* (sponge), *Monosiga brevicollis* (choanoflagellate). Accession numbers are given for outgroup sequences and sequences from model species, for other sequence sources please refer to Supplementary Table S5.

The DnaJ genes have been well catalogued in model species, with the function, expression and subcellular localization of many of their encoded proteins described^13^. Outside model species, however, DnaJ gene diversity is not well described. Hence, to investigate the evolutionary history of *dnajc3* and *dnajc10* we searched the genomes of a representative range of animals, and the genome of a choanoflagellate (the closest unicellular lineage to the animals) for orthologues of *dnajc3*, *dnajc10* and *dnajb9*. We found genes with similar sequence and domain structure in most bilaterian genomes, and in earlier diverging animal Phyla including Porifera, Placozoa, Ctenophore and Cnidaria (Supplementary Table S5). Molecular phylogenetic analyses of these genes also supported their orthology (Fig. 3, Supplementary Fig. S11). We conclude both genes predate the radiation of animals, and hence that orthologues will be found in all animal lineages unless secondarily lost.

## Discussion

Our data reveal that expression levels of canonical HSPs such as *hsp70* and *hsp90*, which are widely used ecological markers of measuring buffering levels, do not predict the level of buffering in *C. intestinalis* embryos. Instead, we found that expression levels of *dnajc3* and *dnjac10* do predict buffering levels. Our genomic survey also showed that orthologues of these genes were found in early diverging Metazoa lineages as well as in the major bilaterian lineages. Importantly, *dnajc3* and *dnajc10* are known as Endoplasmic Reticulum (ER)-associated chaperones^13^. This raises the possibility that ER-associated chaperones act as buffering molecules in embryos across the Metazoa, helping to maintain developmental robustness under fluctuating environmental conditions.

If we assume the correlation between ER DnaJ expression and robustness is indicative of causation, then we can consider the possible underlying molecular mechanisms and how they might relate to canalization. Previous functional analyses of *dnajc3* and *dnajc10* genes have been done in laboratory model species and generally focused on their relation to ER stress. For example, in mice, knockout of *dnajc3* (also known as *p58*^*IPK*^) renders cells more sensitive to ER stress and results in defects in cells involved in a high level of protein secretion^31^,^32^,^33^. Similarly, in the nematode *C. elegans*, a previous study has shown that knockdown of *dnajc3* (known as *dnj-7* in this species) upregulates the expression of a BiP reporter gene, indicating ER stress^34^. Neither of these studies directly examined development, although in *C. elegans* it was reported that *dnajc3* knockdown may lead to reduced fitness as measured by fewer animals reaching the L4 larval stage^33^; this presumably reflects some impact on development, though exactly what was not identified. *dnajc10* (also known as *ERdj5* or *dnj-27*) has been shown to be upregulated by ER stress^35^, and its knockdown by RNA interference in *C. elegans* causes a variety of subcellular defects related to protein homeostasis^36^.

These studies support the idea that *dnajc3* and *dnajc10* are involved in stress resistance, but they do not demonstrate a specific role in the robustness of development to environmental stress. Importantly, contrary to the previous studies described above, levels of developmental buffering do not correlate with the expression of genes that are upregulated by stress (see Fig. 2). This suggests that the ER stress pathways are not directly involved in controlling buffering temperature stress. Recent studies have suggested some chaperones may act in novel ways. For example, Hsp90 prevents mutations by suppressing transposon activity^4^, and can modulate gene regulation by interaction with paused RNA polymerase^37^. Future experiments that explore the role of *dnajc3* and *dnajc10* in embryo robustness should consider the potential for a diversity of mechanisms alongside that of their canonical role in ER protein folding.

Another important finding of our study is that in *C. intestinalis*, the level of buffering was predicted by maternally-inherited expression levels of ER-associated chaperone genes, and not by the conditions in which the embryos were growing. Maternal inheritance of buffering has been previously suggested for sea urchins^38^ and corals^39^, other marine invertebrate species. Since many marine animals broadcast spawn and disperse by planktonic embryos and larvae, maternal supply is likely to be of widespread importance. As limits to species ranges have been shown to reflect the ability to buffer thermal stress^40^, these DnaJ genes may act as better proxies for measuring levels of buffering in embryos than do *hsp83* and *hsp70*. Indeed, this may facilitate the prediction of invasiveness and range change in response to environmental change. This also raises another experimental challenge: to dissect the molecular mechanisms that regulate maternal inheritance of *dnajc3* and *dnajc10* expression. This could be via epigenetic control of zygotic gene expression, or maternal supply of the mRNA. Indeed, even the ER itself is a maternally inherited organelle, although there is no evidence as yet that this is relevant to development. Moreover, it has been shown that eggs in a bryozoan and a marine annelid can be affected by the environment in which the mother resides^41^,^42^. The mechanisms by which ER DnaJs are provided to the developing embryos from the mother in response to environmental variables and how this contributes to phenotypic variability and evolution will be important future tasks for both understanding the evolution of canalization and to predict the impact of global environmental change.

## Methods

### Animals and embryos

Adult *C. intestinalis* of types A and B were collected from either Queen Anne’s Battery or Sutton Harbour in Plymouth, UK, during summers (May to September) in 2010, 2011 and 2012 and kept in tanks under identical conditions and continuous light for 1–2 days until dissection. Animals were fed a mixture of *Rhinomonas reticulate* and *Isochrysis galbana* once a day. Eggs and sperm were dissected from adults and carefully separated before fertilization. Crosses, where the majority of eggs were fertilized, were selected for further experiments and reared at 17 °C until heat shock. Genotypes were identified as previously described^23^,^24^ using sperm samples.

### Heat shock experiments

100 ml of filtered seawater in a crystallizing dish was suspended in a water bath of warm seawater and left to preheat for 1 h. At 8 h post fertilization (when embryos were at the early neurula stage), 500 μl of seawater, containing a few thousand embryos, was transferred to the pre-warmed filtered seawater in crystallizing dishes to an air incubator and kept for 1 h. Additionally, 1 ml of these embryos were collected from the warm seawater at 8 h after fertilization, transferred to fresh filtered seawater in a crystallizing dish at 17 °C and kept in a sealed square plastic box with a lid overnight until the larvae hatched. Numbers of both normal and abnormal larvae were counted after hatching. Unhatched embryos were counted as abnormal larvae. Only batches in which greater than 70% of control embryos hatched normally were used for further statistical analysis. The data were analysed with the statistics package R^43^ using a binomial model, creating a mixed effects model (lmer) fit with the Laplace approximation, with genotype and treatment as fixed effects and random effect of paternal genotype nested by maternal genotype. A *P* value lower than 0.05 was considered as statistically significant. For graphs, we defined normal proportion as: normal proportion = frequency of normal larvae in heat treated section/frequency of normal larvae in control section. Graphs were plotted by box and whisker plot using R^43^.

### RNA isolation

5 ml of filtered seawater containing 1000–2000 developing embryos were collected, dechorionated, washed twice with filtered seawater then immersed in RNA Later (Ambion) within 20 minutes of collection. Samples were stored at 4 °C overnight before long-term storage at −80 °C. RNA was isolated using Trizol (Invitrogen) according to the manufacturer’s instructions. Following chloroform treatment, the supernatant was transferred to a new tube, then mixed with a half volume of High Salt Solution (Takara) and a half volume of isopropanol and incubated at room temperature for 20 minutes to remove polysaccharide contaminations. After centrifuging, RNA pellets were washed once with 70% ethanol, dried at room temperature and dissolved in 100 μl of distilled water and further purified using the RNA micro Purification kit (Qiagen). Genomic DNA contamination was removed by digesting with Dnase I (Qiagen). Quality of the purified RNA was checked using a Bioanalyzer 2100 (Agilent) and only samples that showed a RIN > 8 were used for further analysis.

### cDNA library construction, sequencing and data analysis

RNA was amplified using the MessageAmpIIaRNA kit (Ambion), cDNA synthesized with the cDNA synthesis system (Roche), fragmented and used for cDNA library construction (Library Prep kit Rapid Library Rgt/Adapters, Roche). We chose 454 sequencing for transcriptome analysis, because with the high rate of SNPs between different populations of *C. intestinalis* long reads will increase accuracy of mapping. Sequencing was conducted on one sample from each species type using 454 GS-FLX (Roche), according to the manufacturer’s instructions. This generated 147.56 Mb for type B and 141.98 Mb for type A. All reads were mapped to the KH genome assembly^44^ by GS Reference Mapper command of Newbler version 2.5.3 (Roche) using default parameters. We used BEDTools^45^ to identify gene models where reads are mapped. The standard RPKM expression values^46^ for each KH gene model was calculated. We defined the ratio of expression level as the RPKM value for *C. intestinalis* type B divided by the value for *C. intestinalis* type A.

### qPCR

1 μg of purified total RNA was used for first strand synthesis (SuperScript III First-strand synthesis system for qPCR, Invitrogen), with incubation of 2 h at 55 °C after an initial 30 minutes at 50 °C. The first strand DNA samples were diluted 1/20 for qPCR analysis. Two different primer sets were made for all target loci in the coding region of two different exons where the sequences perfectly matched between types A and B. Prior to qPCR, all primers were checked by PCR observe successful amplification of the target locus in both type A and B samples. In addition, cDNA samples were checked for genomic DNA contamination. Amplified bands were cloned and sequenced to verify correct product amplification. These clones were then diluted and used as standards for calculation of absolute levels of amplification in qPCR. Primer sequences are shown in Supplementary Table 4. Real Time PCRs were performed using Power SYBR Green PCR Master Mix (Applied Biosystems) and a Mastercycler ep realplex (Eppendorf) following the manufacturer’s instructions. Multiple cDNA samples were used as biological replicates to conduct statistical analysis: four heat treated type A embryo RNAs, five heat treated type B embryo RNAs, three control type A embryo RNAs, four control type B embryo RNAs, and multiple hybrid RNAs (four for heat treated AB, three for control AB, six for heat treated BA and four for control BA). Data was plotted as box and whisker plots using R^43^, which accurately shows the nature of data including interquartile range and outliers. The data were analysed by creating a linear mixed effects models of treatment and genotype as fixed effects and RNA origin nested by primer sets as random effect, fit by Maximum Likelihood using R^43^. For Bonferroni correction, a *P-*value of 0.0045 or less was taken as statistically significant. Correlation between normal proportion and expression levels was assessed by creating fixed effect linear models. The model giving a smaller *P*-value was chosen, where a *P-*value of 0.025 or less was taken as statistically significant (Bonferroni correction of normal criteria 0.05 divided by the primer sets of 2).

### *dnajc3* and *dnajc10* ortholog identification

Databases (Supplementary Table S5) were searched with human DNAJC3 or DNAJC10 amino acid sequences, using BlastP against filtered gene models where possible, or TBlastN against genomic contigs where models were not available. Top hits were searched back against metazoan nucleotide data using TBlastN. In most cases this hit back to the original query gene from multiple species, with exceptions including the top hits for DNAJC10 in *D. melanogaster*, *T. adhaerens* and *A queenslandica* (Supplementary Table S5). Since a clear *dnajc10* orthologue was identified in a unicellular outgroup to the animals, *M. brevicollis*, this either reflects gene loss or missing data in these species.

### Molecular phylogenetics

DnaJ domain sequences were aligned using ClustalW as part of BioEdit^47^ and checked by eye for appropriate alignment. Bayesian analysis was undertaken using MrBayes 3.1^48^ with the amino acid model set to mixed and other parameters as default. One million generations were performed, with the first 25% discarded when compiling summary trees and statistics. Maximum Likelihood analysis was conducted in MEGA 6^49^, using the JTT model and 100 bootstrap replicates.

## Additional Information

**How to cite this article**: Sato, A. *et al.* Molecular basis of canalization in an ascidian species complex adapted to different thermal conditions. *Sci. Rep.* **5**, 16717; doi: 10.1038/srep16717 (2015).

## Supplementary Material

Supplementary material

Supplementary Table 1
