# Supplementary material for: Molecular basis of canalization in an ascidian species complex adapted to different thermal conditions
Source: Sci Rep. 2015 Nov 18;5:16717. doi: 10.1038/srep16717 (PMC4649386; doi:10.1038/srep16717)
Supplement: Supplementary material [file srep16717-s1.pdf]

Supporting online material for:

Molecular basis of canalization in an ascidian species complex adapted to different thermal conditions

Atsuko Sato, Takeshi Kawashima, Manabu Fujie, Samantha Hughes, Nori Satoh, Sebastian M Shimeld

To whom correspondence should be addressed to: Atsuko Sato (email: pterobranch@gmail.com)

This PDF file includes:

Tables: S1-S5

Figs: S1-11

Supplementary Table S1. Complete list of gene models found in the transcriptome analysis. (see separate PDF file)

Supplementary Table S2. The complete list of chaperone and other molecules found in the transcriptome analysis. Raw counts ('B count' and 'A count') were normalized by the RPKM method [46]. Those genes validated by qPCR are highlighted in blue. We did not evaluate genes with less than 10 counts in either species, and others were not validated as we were not able to develop effective qPCR primers. Gene models derive from the KH gene model set [15]. Note some genes are represented by more than one gene model, reflecting splice variants and/or uncertainty in the modeling process.

Gene name	KH Gene model	Gene Model length (bp)	B count	A count	B normalised	A normalised	B/A ratio
dnajc4	KH.C4.524.v1.A.ND1-1	827	1	7	2.57	18.60	0.14
methuselah	KH.C1.1280.v1.A.ND1-1	3642	1	4	0.58	2.41	0.24
dnajc12	KH.C2.472.v1.A.SL1-1	635	2	6	6.70	20.77	0.32
dnajb9	KH.C1.914.v1.A.ND1-1	1565	11	32	14.95	44.94	0.33
rbj	KH.C2.432.v1.A.ND1-1	1039	22	48	45.03	101.53	0.44
*dnajc*13	KH.C9.314.v1.A.SL1-1	7294	8	17	2.33	5.12	0.46
dnajc3	KH.C1.269.v1.A.SL1-1	1831	16	30	18.58	36.01	0.52
dnajc7	KH.C9.428.v1.A.ND2-1	882	10	18	24.11	44.85	0.54
dnajc7	KH.C9.428.v2.A.ND3-1	1950	25	44	27.26	49.59	0.55
hspa9b	KH.C3.429.v1.A.SL2-1	2290	237	401	220.09	384.85	0.57
hspa9b	KH.C3.429.v1.A.SL1-1	2278	237	401	221.25	386.87	0.57
hsp83	KH.C3.148.v2.C.ND2-1	1718	1383	2269	1711.94	2902.61	0.59
dnajc7	KH.C9.428.v3.A.SL1-1	2104	30	49	30.32	51.18	0.59
hsp83	KH.C3.148.v1.C.SL1-1	2169	1494	2417	1464.80	2449.03	0.60
hsp83	KH.C3.148.v1.C.SL3-1	2187	1494	2417	1452.75	2428.87	0.60
ubadc1	KH.L133.5.v1.A.SL3-1	1305	17	26	27.70	43.79	0.63
ubadc1	KH.L133.5.v1.A.SL1-1	1206	17	26	29.98	47.38	0.63
ubadc1	KH.L133.5.v1.A.ND2-1	1388	17	26	26.05	41.17	0.63
gng10	KH.C8.810.v1.A.SL1-1	1622	15	22	19.67	29.81	0.66
gng10	KH.C8.810.v1.A.ND2-1	1630	15	22	19.57	29.66	0.66
gng10	KH.C8.810.v1.A.SL3-1	1625	15	22	19.63	29.75	0.66
rbj	KH.C2.282.v1.A.ND1-1	1835	43	63	49.83	75.45	0.66
rbj	KH.C2.282.v2.A.ND1-1	1917	43	63	47.70	72.23	0.66
dnajc10	KH.C9.567.v2.A.SL1-1	2711	74	107	58.05	86.74	0.67
trap1	KH.C14.318.v1.A.ND1-1	2739	64	92	49.69	73.82	0.67
bat3	KH.L22.22.v1.B.SL1-1	3638	46	62	26.89	37.45	0.72
tcp1zeta	KH.S1665.1.v1.A.SL1-1	1796	87	117	103.02	143.17	0.72
dnajc8	KH.C14.114.v1.A.SL1-1	1260	6	8	10.13	13.95	0.73
dnajc5	KH.C2.944.v2.A.SL1-1	1682	120	158	151.72	206.45	0.73
dnajc5	KH.C2.944.v1.A.SL1-1	1596	120	158	159.90	217.57	0.73
tcp1theta	KH.L153.45.v1.A.SL1-1	2043	142	181	147.81	194.71	0.76

tcp1theta	KH.L153.45.v2.A.SL1-1	1894	142	181	159.44	210.03	0.76
tcp1theta	KH.L153.45.v1.A.ND2-1	2051	142	181	147.24	193.95	0.76
bag1	KH.C3.610.v2.A.ND1-2	2529	9	11	7.57	9.56	0.79
ubqln	KH.C4.426.v1.A.SL2-1	2283	93	110	86.63	105.89	0.82
ubqln	KH.C4.426.v1.A.SL1-1	2252	93	110	87.82	107.35	0.82
grpel	KH.C8.114.v1.A.SL1-1	897	39	46	92.46	112.70	0.82
timm44	KH.C8.96.v1.A.ND2-1	1479	29	34	41.70	50.52	0.83
timm44	KH.C8.96.v1.A.SL1-1	1468	29	34	42.01	50.90	0.83
hspa8	KH.L141.53.v1.A.ND1-1	2288	3437	4018	3194.57	3859.50	0.83
hspa8	KH.L141.53.v1.A.SL2-1	2254	3437	4017	3242.76	3916.74	0.83
hspa8	KH.L141.53.v1.A.SL3-1	2240	3437	4017	3263.02	3941.22	0.83
dnajc10	KH.C9.567.v1.A.ND2-1	707	18	21	54.14	65.28	0.83
hsp60	KH.C6.85.v1.A.SL1-1	2703	353	409	277.73	332.55	0.84
dnajb11	KH.C9.96.v1.A.SL2-1	1376	133	149	205.55	237.98	0.86
dnajb11	KH.C9.96.v1.A.SL1-1	1363	133	149	207.51	240.25	0.86
rbj	KH.C2.331.v1.A.SL1-1	1712	26	29	32.30	37.23	0.87
rbj	KH.C2.331.v1.A.SL2-1	1715	26	29	32.24	37.16	0.87
wbscr18	KH.C7.436.v2.A.SL2-1	928	63	70	144.37	165.78	0.87
wbscr18	KH.C7.436.v2.A.SL3-1	962	63	70	139.27	159.92	0.87
rbj	KH.C2.13.v1.A.SL1-1	1197	28	31	49.75	56.92	0.87
wbscr18	KH.C7.436.v1.A.ND1-1	1075	64	70	126.61	143.11	0.88
hsp90b1	KH.L61.14.v1.A.SL1-1	2970	116	120	83.06	88.80	0.94
dnajb12/14/dnajc18	KH.C1.303.v1.A.ND1-1	2490	102	99	87.11	87.38	1.00
dnaja1/4	KH.C3.139.v1.C.SL1-1	1948	616	594	672.48	670.15	1.00
dnaja1/4	KH.C3.139.v1.C.SL4-1	1951	616	594	671.45	669.12	1.00
hspa5/hsc70-3	KH.C9.680.v1.A.SL1-1	2382	124	119	110.71	109.79	1.01
dnaja1/4	KH.C3.139.v2.C.SL2-1	1235	558	534	960.85	950.28	1.01
dnaja1/4	KH.C3.139.v3.C.ND3-1	1110	519	492	994.34	974.14	1.02
hyou	KH.C11.193.v1.A.SL1-1	3332	87	80	55.53	52.77	1.05
bag3	KH.C8.859.v1.A.nonSL1-1	957	176	157	391.10	360.55	1.08
dnaja3	KH.C14.339.v1.B.ND1-1	1924	22	19	24.32	21.70	1.12
dnajc7	KH.C9.410.v1.C.ND1-1	1508	290	248	408.96	361.43	1.13
dnajc7	KH.C9.410.v1.C.nonSL2-1	900	290	248	685.24	605.60	1.13
dnajc6	KH.C8.356.v2.A.SL1-1	4610	125	106	57.66	50.53	1.14
HSPH/*hspa4*	KH.C14.180.v1.A.SL1-1	3053	504	422	351.07	303.78	1.16
HSPH/*hspa4*	KH.C14.180.v2.A.ND2-1	2884	503	421	370.90	320.82	1.16
dnajc2	KH.C1.289.v1.A.ND1-1	2065	159	128	163.74	136.23	1.20
sec63	KH.S437.4.v1.A.SL1-1	2415	167	132	147.06	120.13	1.22
dnajc6	KH.C8.356.v1.A.ND4-1	4225	122	96	61.41	49.94	1.23
dnajc6	KH.C8.356.v1.A.SL3-1	3796	122	96	68.35	55.58	1.23
dnajc6	KH.C8.356.v1.A.SL2-1	3859	122	96	67.23	54.67	1.23
dnajc11	KH.C4.798.v1.A.SL1-1	2190	21	16	20.39	16.06	1.27
Magmas	KH.C2.716.v2.A.ND2-1	1101	25	19	48.29	37.93	1.27
dnajb13	KH.L4.20.v1.C.SL2-1	862	4	3	9.87	7.65	1.29
hsc20	KH.C10.111.v1.A.SL1-1	1000	4	3	8.51	6.59	1.29
dnajb13	KH.L4.20.v1.A.ND1-1	1374	4	3	6.19	4.80	1.29
tcpbeta	KH.C14.531.v1.A.SL1-1	1798	132	94	156.13	114.90	1.36
tcpeta	KH.S1300.1.v1.A.nonSL1-1	1885	95	65	107.18	75.78	1.41
dnajc9	KH.C12.582.v1.C.SL1-1	1061	3	2	6.01	4.14	1.45
tcp1epsilon	KH.L95.8.v1.A.ND1-1	1802	229	150	270.25	182.94	1.48

tcp1epsilon	KH.L95.20.v1.A.ND1-1	1839	229	150	264.81	179.26	1.48
tcp1alpha	KH.C5.523.v1.A.SL1-1	2065	228	148	234.80	157.51	1.49
tcp1alpha	KH.L96.2.v1.A.ND2-1	1996	251	161	267.42	177.27	1.51
tcp1alpha	KH.L96.2.v1.A.SL1-1	1992	251	161	267.96	177.63	1.51
dnajc1	KH.L112.3.v1.A.ND1-1	1689	12	7	15.11	9.11	1.66
dnajc1	KH.L112.3.v1.A.SL2-1	1657	12	7	15.40	9.28	1.66
dnajb2/3/6/7/8	KH.C2.518.v1.A.SL1-1	1437	315	178	466.17	272.23	1.71
dnajb2/3/6/7/8	KH.C2.518.v1.A.SL2-1	1512	316	178	444.45	258.73	1.72
danjb1/4/5	KH.L170.59.v1.A.ND1-1	1595	1235	639	1646.63	880.48	1.87
bag3	KH.C8.85.v2.A.ND3-2	1043	256	121	521.97	254.96	2.05
bag3	KH.C8.85.v1.A.SL1-2	805	280	119	739.69	324.88	2.28
HSPB	KH.S455.4.v1.A.SL1-1	1100	72	29	139.20	57.94	2.40
dnajc16	KH.L20.7.v1.A.SL1-1	2554	11	4	9.16	3.44	2.66
hsp70	KH.L46.6.v1.A.ND1-1	1768	3433	1130	4129.34	1404.67	2.94
bag3	KH.C8.85.v5.A.ND2-1	1605	1600	478	2119.99	654.53	3.24
bag3	KH.C8.85.v4.A.SL1-1	1468	1627	478	2356.95	715.61	3.29
SIL (BAP)	KH.L124.16.v1.A.ND1-1	1828	31	7	36.06	8.42	4.29

Supplementary Table S3. qPCR analysis of prioritized chaperone genes including *dnajc3* and *dnajc10*. Two independent sets of primers designed on different exons were used for each gene, except where only one functional primer set could be established (*hspa9b* and *hspa8*). AA heat/ BB heat compares type A conspecific crosses with type B conspecific crosses after heat shock at 27ºC for 1h. The data show significant differences in *dnajc3*, *tcp1theta*[50] and *trap1*, with marginal effect in *dnajc10* and *hsp83* but not in *dnajc5*, *hsp60*, *hsp70*, *hspa8* and *hsp9b*. AA (heat/cont) shows comparison between heat shocked and control expression levels in type A conspecific crosses; BB (heat/cont) shows comparison between heat shocked and control expression levels in type B conspecific crosses. The data confirmed induction of transcription by heat shock of *hsp60*, *hsp70*, and *hspa8* in at least one of these species. AB heat/ BA heat shows comparison of alternative hybrid crosses (AB; type A eggs and type B sperm. BA; type B eggs and type A sperm). Those not induced by heat, such as *dnajc3, dnajc10, dnajc5, tcp1theta* and *trap1* (and marginally) *dnajb9)*, show maternal inheritance of the expression level. A positive correlation to thermal tolerance was only observed for *dnajc3 and dnajc10* (Fig. 2). *P*-value below the Bonferroni-corrected significance threshold of 0.0045 are shown in green, and $P <$ 0.01 is considered as marginally significant and shown in blue.

Target	Primers	B/A 454 reads ratio	AA cont/ BB cont	AA heat/ BB heat	AA (heat/cont)	BB (heat/cont)	AB heat/ BA heat
dnajb9	1 + 2	0.33	0.0103	0.2654	0.4767	0.4142	0.0061
dnajc10	1 + 2	0.67	0.0067	0.0067	0.1	0.1	<0.0001
dnajc3	1 + 2	0.51	2.00E-04	2.00E-04	0.1297	0.1297	<0.0001
dnajc5	1 + 2	0.73	0.0029	0.4388	0.2101	0.2562	0.0023
hsp60	1 + 2	0.83	1.00E-04	0.0116	0.1772	0.0016	0.2526
hsp70	1 + 2	2.94	0.4683	0.4683	<0.0001	<0.0001	0.0278
hsp83	1 + 2	0.59	0.0081	0.0081	0.013	0.013	0.8541
hspa8	1	0.83	0.0882	0.0882	0.0021	0.0021	0.8298
hspa9b	1	0.57	0.0366	0.0366	0.048	0.048	0.0066
TCP1theta	1 + 2	0.76	<0.0001	<0.0001	0.0331	0.0331	8.00E-04
trap1	1 + 2	0.67	1.00E-04	1.00E-04	0.6431	0.6431	1.00E-04

Supplementary Table S4. List of primer sequences used for qPCR analysis.

Target	Primer sets		Sequence	Target length
dnajc3	1	DNAJC3-F3	AGCAGCAAAGAAGGTCATGC	106
		DNAJC3-R3	ATTTCCACGTTTAATGCGCC	
	2	DNAJC3-F4	CAGCCTTGCCGGCCACTGTG	114
		DNAJC3-R4	GGAATCCAGGAAGGCACGAA	
dnajc10	1	DNAJC10-F1	CAGGATCCTGATGAAATAAC	204
		DNAJC10-R1	GCGACAATACTTCCGCAACA	
	2	DNAJC10-F2	GCCCGTGTATGGCATTCTCA	117
		DNAJC10-R2	ACCCCAGCTTGGCTACAAAT	
hsp83	1	Hsp90-F5	GGGTTCCTTCAATATCACTC	127
		Hsp83-5R	AACCTGGTCTCTTGATTCAC	
	2	Hsp83-6F	ATGAGCTTGATCATCAACAC	92
		Hsp83-6R	TATCGGATCTTGTCCAAGGC	
hsp60	1	Hsp60-3F	AACTTGCAAGTGGTGTTGCTG	111
		Hsp60-3R	CTACTGCAGCTCGTGTTGCG	
	2	Hsp60_F5	CGCTGGCATGGAAGGACAAC	98
		Hsp60_R5	GTCGTAGCCATAGTCACCTT	
hsp70	1	Hsp70_F5	CACTGGCCTTTCAAGGTAGT	229
		Hsp70_R5	CCAGCCAAGTTCTTATCGAG	
	2	Hsp70-F3	CCATTCGGTTATCGAAGTCC	237
		Hsp70_R4	ATCCTGGTTGATCCTCCGAC	
trap	1	trap F2	CAACTTGAGCAGAGAACTGC	107
		trap R2	GGTCGCGACGACTTTGATCC	
	2	trap1_F5	TGAACACGAGTTTCAAGCTG	75
		trap1_R5	TCTAACGCGTCACTCGCATT	
dnajc5	1	DNAJC5-F1	AATGAACCTCAACCTCAAGG	113
		DNAJC5-R1	GTCCTCAGGTTGTCTTCTCC	
	2	DNAJC5-F2	CCCTTGGGCGCATCACCTCC	121
		DNAJC5-R2	CGACAACTATGATTTATGCA	
tcp1theta	1	TCP1theta_F1	CGTAGATAACATTCGTGTGA	139
		TCP1theta_R1	GACAAGTGTATACCGCTACT	
	2	TPC1theta_F2	GGAGAGGGCAGTAGATGACG	101
		TCPtheta_R2	CTTTTCGCGAGTTCTATCTC	
dnajb9	1	DNAJB9-F3	GAGCCAGACGCGGAAGCAA	118
		DNAJB9-R3	CCCATATTGTCGTTCAAACC	
	2	DNAJB9-F4	CCGACTTCCAAAGTGACGAC	90
		DNAJB9-R4	GATGTGAATGGGAACCTTCC	
hspa9b	1	HSPA9B-F5	TTATAGCCGTGTACGACCTT	192
		HSPA9B-R5	GGGCCGCTTGCATCCATGGT	
hspa8	1	HSPA2/8-4F	CTTCGTGACGCTAAGATGGG	91
		HSPA2/8-4R	GGAATACGAGTGGAGCCACC	

Supplementary Table S5. ER chaperones identified from genomes of various organisms with special focus on marine invertebrates. All except *M. brevicollis* are animals, and most have planktonic larvae. Accession numbers are given where these exist. In other cases gene models or equivalent identifiers are listed, which can be accessed via the database shown.

**Species**	**Database/ Source**	***dnajc3* orthologue**	***dnajc10* orthologue**	***dnajb9* orthologue**
***H. sapiens* (human)**	NCBI	EAX08959	AAI17300	Q9UBS3
***C. intestinalis* (sea squirt)**	Aniseed	KH.C1.269.	KH.C9.567.	KH.C9.914.
***B. floridae* (amphioxus)**	JGI	jgi|Brafl1|122581| jgi|Brafl1|119688|	jgi|Brafl1|125408|estExt_fgenesh2_pg.C_1820034	jgi|Brafl1|203558|e_gw.10.429.1
***S. purpuratus* (sea urchin)**	Spbase	SPU_005174	SPU_017259	Not found
***S. kowalevskii* (acorn worm)**	NCBI; http://blast.hgsc.bcm.tmc.edu/	Contig96435*	XP_002740489.1	XP_002730538.1
***C. teleta* (annelid)**	JGI	jgi|Capca1|228061|estExt_fgenesh1_pg.C_660051	jgi|Capca1|228052|estExt_fgenesh1_pg.C_660035	jgi|Capca1|19566|estExt_fgenesh1_pm.C_500003 (DNAJB1/4/5 and DNAJB9 orthologue) jgi|Capca1|225634|
L. gigantea (limpet)	JGI	jgi|Lotgi1|151060|fgenesh2_kg.C_sca_80000004	jgi|Lotgi1|171701| jgi|Lotgi1|237821|	jgi|Lotgi1|235906| jgi|Lotgi1|168037|
***C. gigas* (oyster)**	NCBI	EKC24329	EKC28571	EKC30878.1
***C. elegans* (nematode)**	wormbase	*dnj7*	*dnj27*	Not found
***D. melanogaster* (fruitfly)**	flybase	P58IPK	Not found	Not found
***A. digitifera* (coral)**	http://marinegenomics.oist.jp/genomes/gallery	aug_v2a.19366.t1*	aug_v2a.21205.t1	aug_v2a.18536.t1
***N. vectensis* (sea anemone)**	JGI	jgi|Nemve1|174406|estExt_gwp.C_3040037	jgi|Nemve1|163820|estExt_gwp.C_440026	jgi|Nemve1|106836|e_gw.84.88.1
T. adhaerens	JGI	jgi|Triad1|32828|e_	Not found	jgi|Triad1|56372|fgenesh

**(placozoan)**		gw1.26.21.1	Triad1|60480?	TA2_pg.C_scaffold_5000266
***M. leidyi* (ctenophore)**	http://research.nhgri.nih.gov/mnemiopsis/	ML06971a	ML01344a	ML07989a<br>ML37595a<br>ML01344a
**A. *queenslandica* (sponge)**	NCBI	XP_003390240.1<br>XP_003391066.1<br>XP_003389230.1	Not found	Not found
***M. brevicollis* (choanoflagell-ate)**	JGI	jgi|Monbr1|6406| jgi|Monbr1|15161| jgi|Monbr1|18047| jgi|Monbr1|18048| jgi|Monbr1|20049|	jgi|Monbr1|21911|	Not found<br>jgi|Monbr1|22976?

*Only whole genome contig and/or incomplete gene prediction available. Orthology of hit verified by reciprocal BLAST.

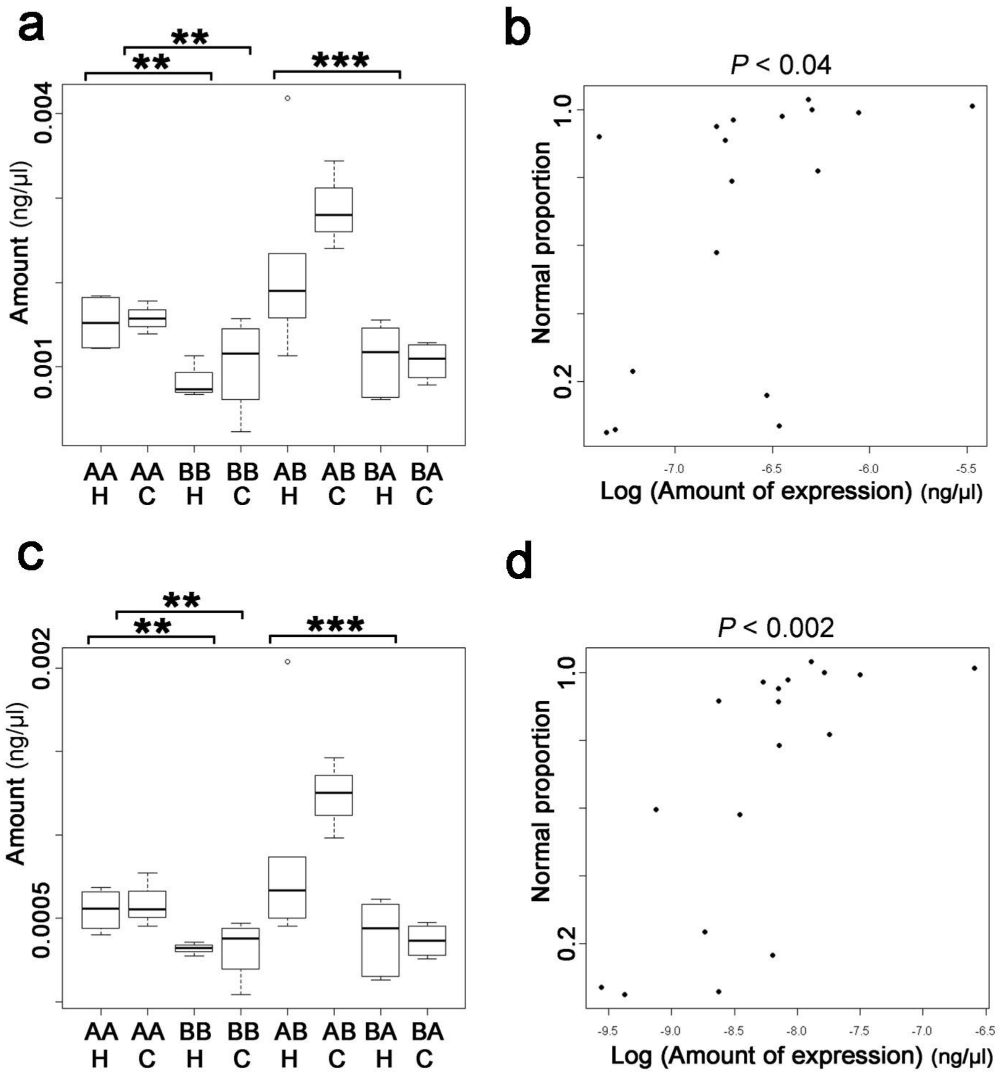

Supplementary Fig. 1. Comparison of expression levels of *dnajc3*. a, b, show results from primer set 1, whereas **c**, **d** show primer set 2. **a, c,** Quantification of *dnajc3* expression levels by qPCR in control (C) or heat shocked (H: 27ºC for 1h at neurula stage as in Fig. 1a) embryos. Heat shock did not significantly affect the expression of *dnajc3*, however expression in type A (AA) was significantly higher than in type B (BB) (P = 0.002). Reciprocal hybridization (AB/BA) showed strong correlation to the maternal genotype (P < 0.0001). Significances are shown by asterisks: ***P* <0.01; ****P* <0.001. **b, d,** Correlation between the amount of *dnajc3* transcripts and Normal proportion after heat shock in individual crosses. Analysis of Variance (ANOVA) rejected the null hypothesis and supported linear correlation of the amount of *dnajc3* transcripts and normal proportion (P < 0.04 for primer sets 1 and P < 0.002 for primer sets 2).

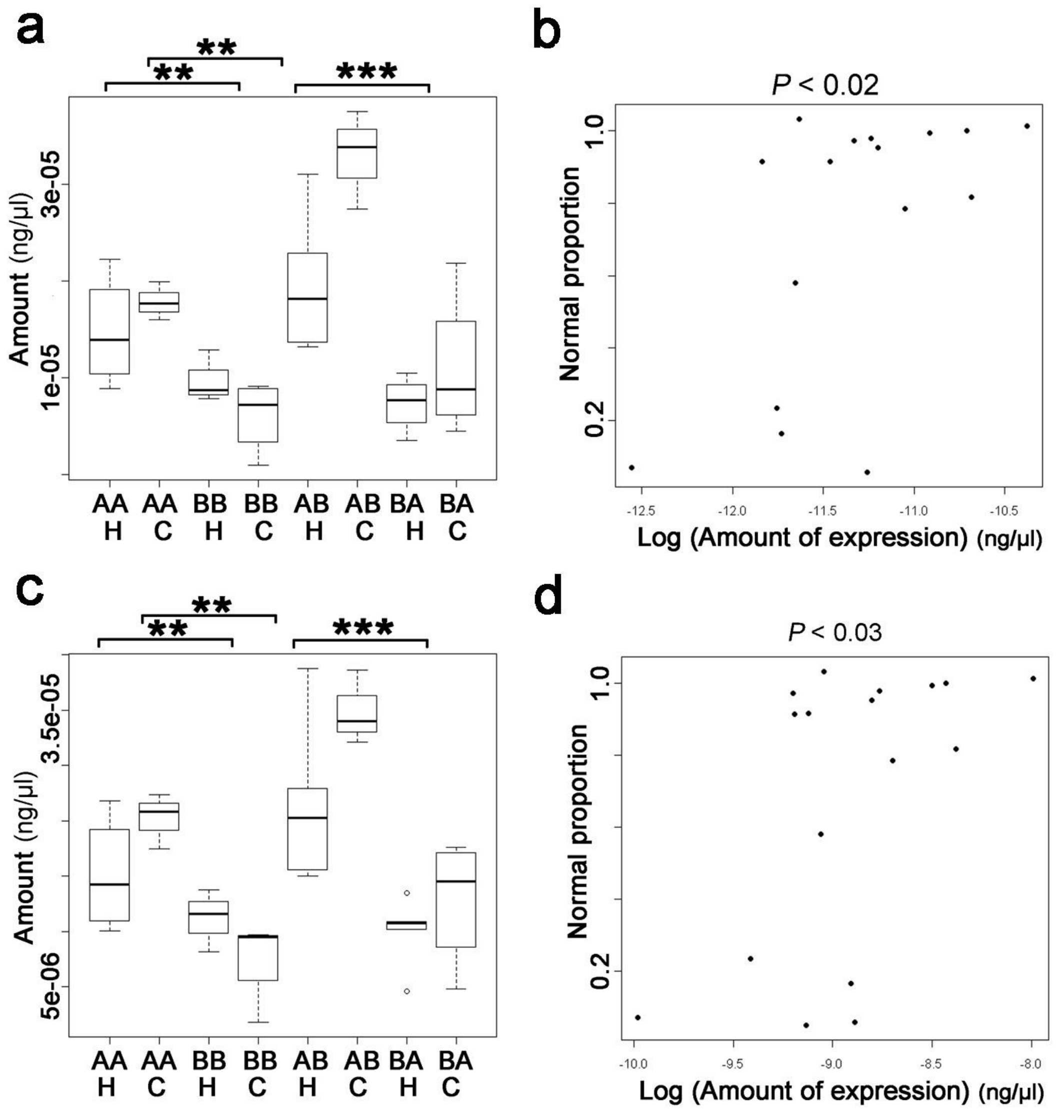

Supplementary Fig. 2. Comparison of expression levels of *dnajc10*. a, b, show results from primer set 1, whereas **c**, **d** show primer set 2. **a, c,** Quantification of *dnajc10* expression levels by qPCR in control (C) or heat shocked (H: 27ºC for 1h at neurula stage as in Fig. 1a) embryos. Heat shock did not significantly affect the expression of *dnajc10*, and expression in type A (AA) under control condition was significantly higher than in type B (BB) (P = 0.0067). Reciprocal hybridization (AB/BA) suggested this was linked to the maternal genotype (P << 0.0001). Significances are shown by asterisks: ** P <0.01; *** P <0.001. **b, d,** Correlation between the amount of *dnajc10* transcripts and normal proportion after heat shock in individual crosses. Analysis of Variance (ANOVA) supports linear correlation of the amount of *dnajc10* transcripts and Normal proportion (P = 0.0197 for primer set 1 and P = 0.02665 for primer set 2).

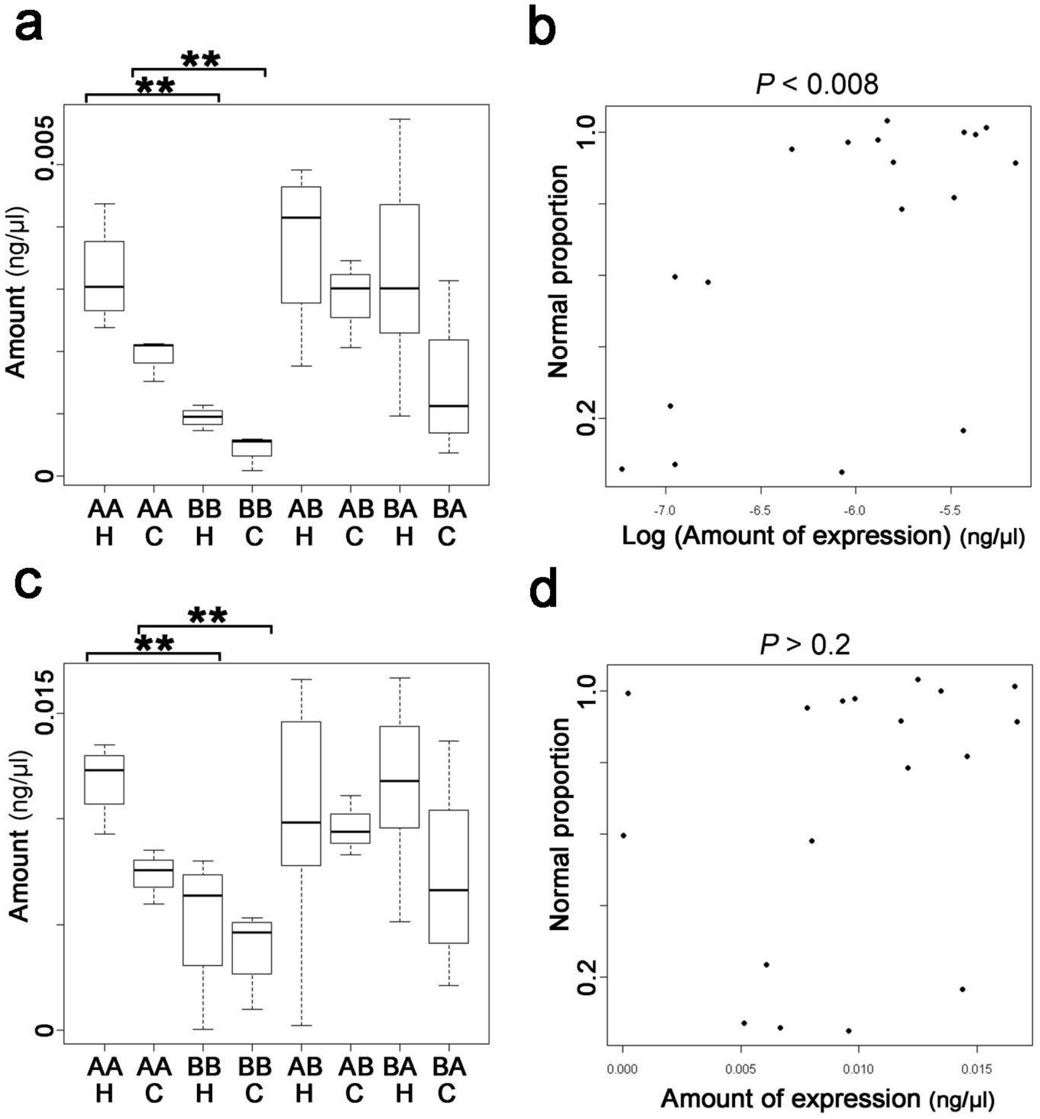

Supplementary Fig. 3. Comparison of expression levels of *hspa83*. a, b, show results from primer set 1, whereas **c**, **d** show primer set 2. **a, c,** Quantification of *hspa83* expression levels by qPCR in control (C) or heat shocked (H: 27ºC for 1h at neurula stage as in Fig. 1a) embryos. Heat shock did not significantly affect the expression of *hspa83* (P = 0.013), but expression in type A (AA) was significantly higher than in type B (BB) (P = 0.0081). However, reciprocal hybridization (AB/BA) did not suggest this was linked to the maternal genotype (P > 0.85). Significances are shown by asterisks: ** P <0.01. **b, d,** Correlation between the amount of *hspa83* transcripts and normal proportion after heat shock in individual crosses. Analysis of Variance (ANOVA) did not support linear correlation of the amount of *hsp83* transcripts and Normal proportion (P < 0.008 for primer set 1; for primer set 2 P > 0.25).

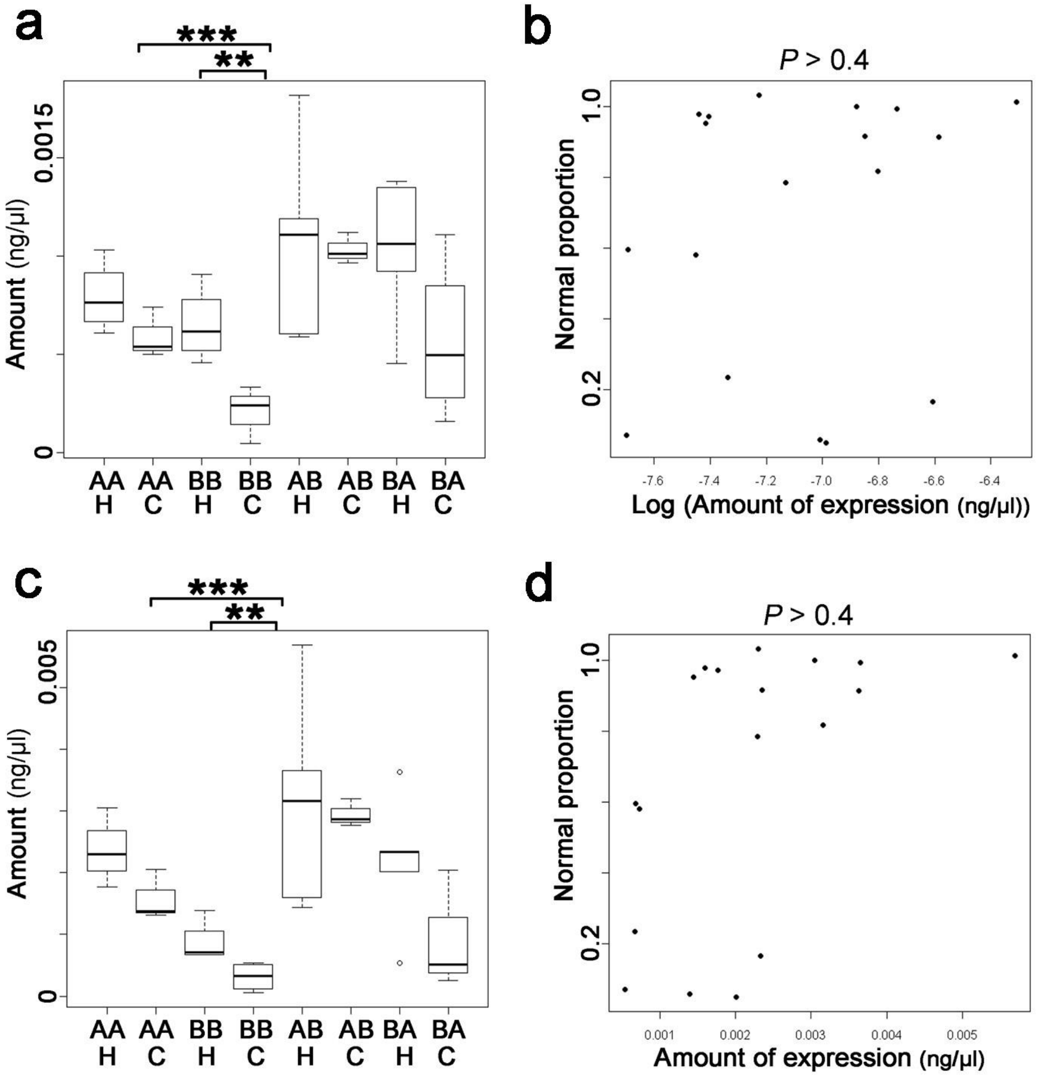

Supplementary Fig. 4. Comparison of expression levels of *hsp60*. a, b, show results from primer set 1, whereas **c**, **d** show primer set 2. **a, c,** Quantification of *hsp60* expression levels by qPCR in control (C) or heat shocked (H: 27ºC for 1h at neurula stage as in Fig. 1a) embryos. Heat shock significantly affected the expression of *hsp60* only in type B (BB). Comparisons of expression in type A (AA) and in type B (BB) showed significant differences under control condition (P = 0.001). However, reciprocal hybridization (AB/BA) did not suggest this was linked to the maternal genotype (P = 0.2526). Significances are shown by asterisks: ** P <0.01; ***P <0.001. **b, d,** Correlation between the amount of *hsp60* transcripts and Normal proportion after heat shock in individual crosses. Analysis of Variance (ANOVA) did not support linear correlation of the amount of *hsp60* transcripts and normal proportion (P > 0.42 for both primer sets 1 and 2).

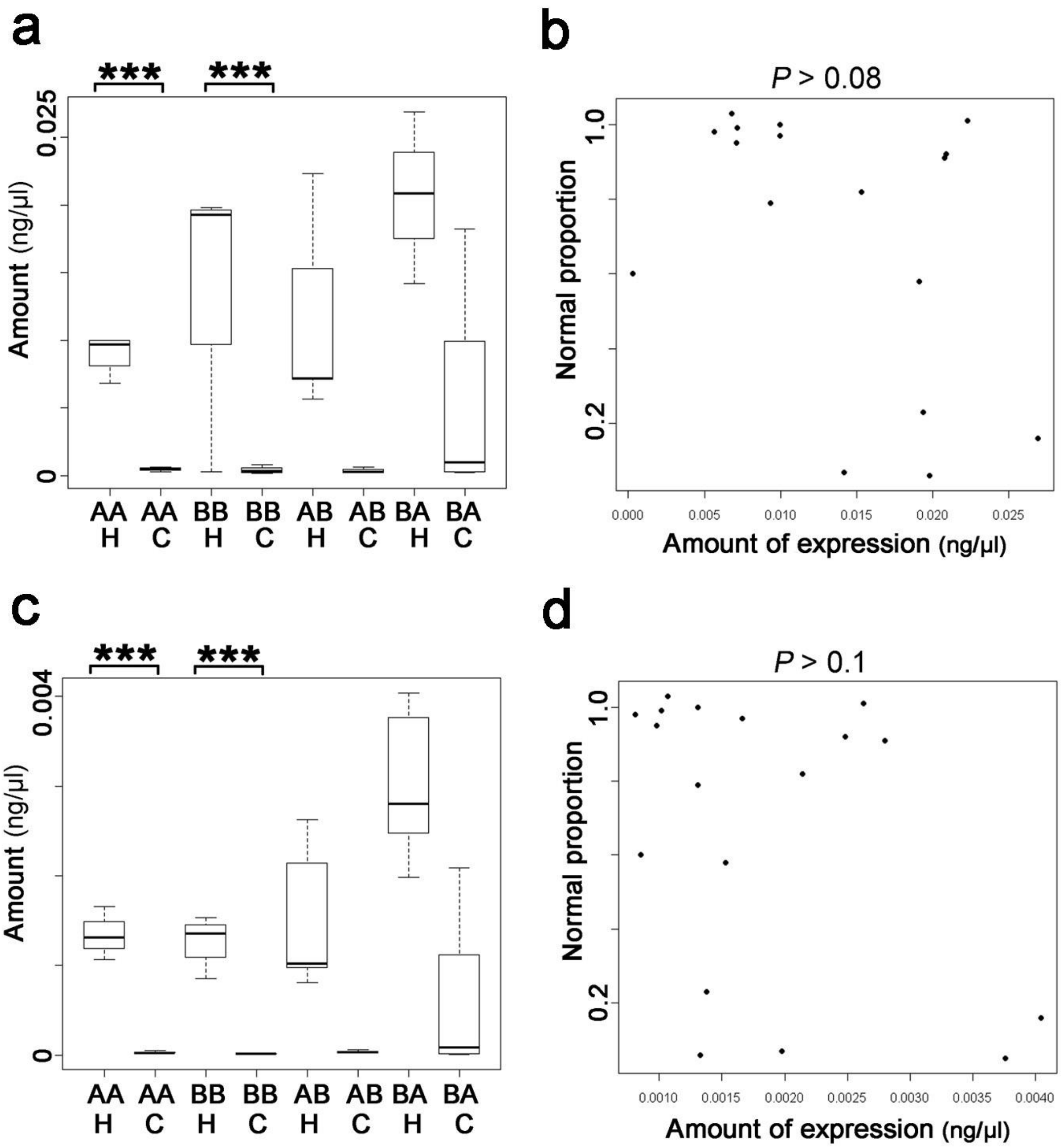

Supplementary Fig. 5. Comparison of expression levels of *hsp70*. a, b, show results from primer set 1, whereas **c**, **d** show primer set 2. **a, c,** Quantification of *hsp70* expression levels by qPCR in control (C) or heat shocked (H: 27ºC for 1h at neurula stage as in Fig. 1a) embryos. Heat shock significantly affected the expression of *hsp70* in both types A and B ($P << 0.0001$). Reciprocal hybridization (AB/BA) did not show its link to the maternal genotype ($P > 0.02$), but the level of expression was negatively correlated to thermal tolerance of these hybrid types. Significances are shown by asterisks: ****P* <0.001. **b, d,** Correlation between the amount of *hsp70* transcripts and normal proportion after heat shock in individual crosses. Analysis of Variance (ANOVA) did not support linear correlation of the amount of *hsp70* transcripts and Normal proportion ($P = 0.08933$ for primer set 1 and $P = 0.103$ for primer set 2).

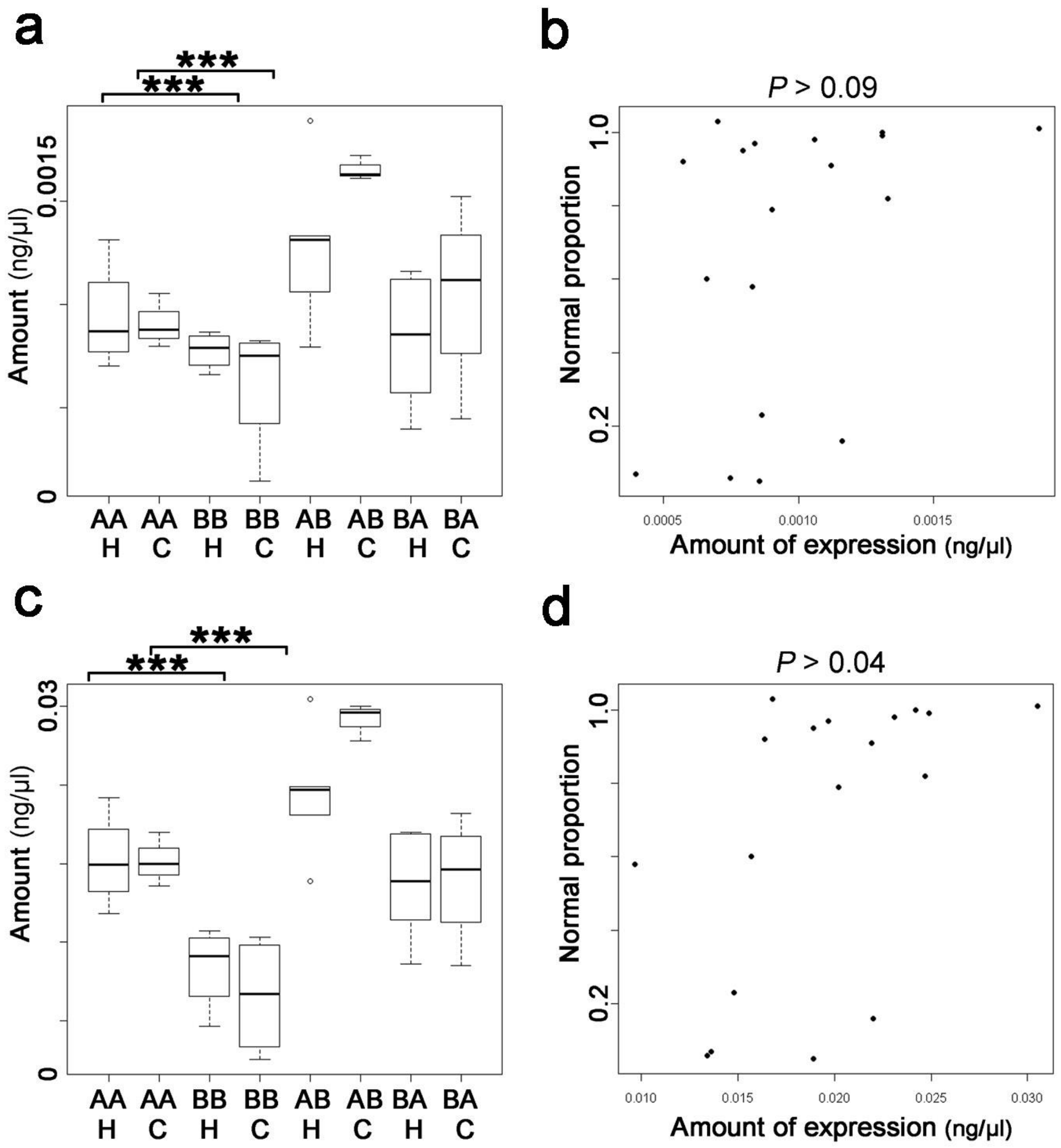

Supplementary Fig. 6. Comparison of expression levels of *trap1*. a, b, show results from primer set 1, whereas **c**, **d** show primer set 2. **a, c,** Quantification of *trap1* expression levels by qPCR in control (C) or heat shocked (H: 27ºC for 1h at neurula stage as in Fig. 1a) embryos. Heat shock did not significantly affect the expression of *trap1* (P = 0.6431), but expression in type A (AA) was significantly higher than in type B (BB) ($P \ll 0.0001$). Reciprocal hybridization (AB/BA) also showed this was linked to the maternal genotype ($P \ll 0.0001$). Significances are shown by asterisks: ***P <0.001. **b, d,** Correlation between the amount of *trap1* transcripts and normal proportion after heat shock in individual crosses. Analysis of Variance (ANOVA) did not supports linear correlation of the amount of *trap1* transcripts and Normal proportion (P = 0.09315 for primer set 1 and P = 0.046 for primer set 2).

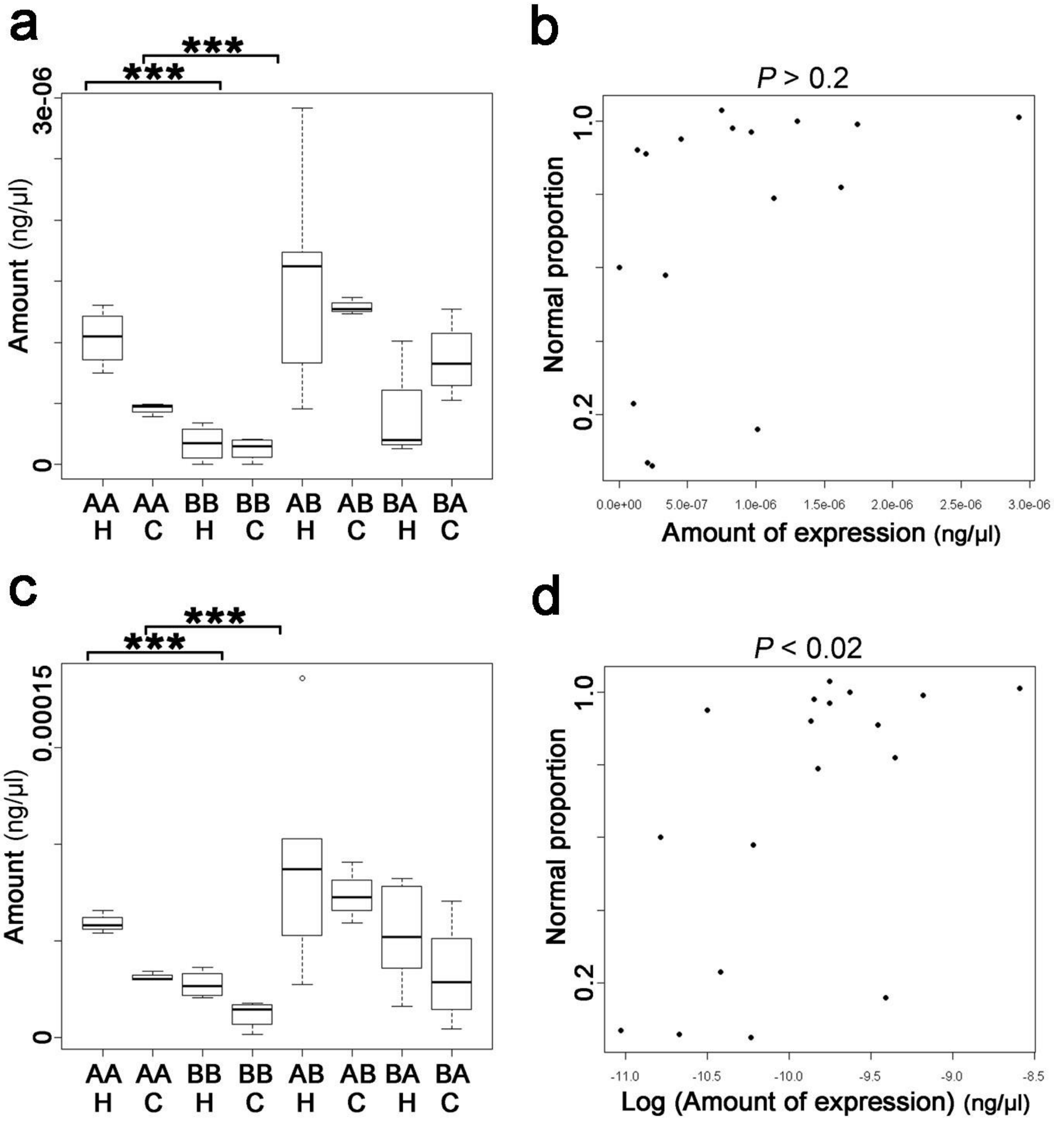

Supplementary Fig. 7. Comparison of expression levels of *tcp1theta*. a, b, show results from primer set 1, whereas **c**, **d** show primer set 2. **a, c,** Quantification of *trap1* expression levels by qPCR in control (C) or heat shocked (H: 27ºC for 1h at neurula stage as in Fig. 1a) embryos. Heat shock did not significantly affect the expression of *TCP1theta* ($P = 0.0331$), but expression in type A (AA) was significantly higher than in type B (BB) ($P << 0.0001$). Reciprocal hybridization (AB/BA) also suggested this was linked to the maternal genotype ($P = 0.0008$). Significances are shown by asterisks: ***$P < 0.001$. **b, d,** Correlation between the amount of *trap1* transcripts and Normal proportion after heat shock in individual crosses. Analysis of Variance (ANOVA) did not supports linear correlation of the amount of *tcp1theta* transcripts and normal proportion (P=0.286 for primer set 1 and P=0.6196 for primer set 2).

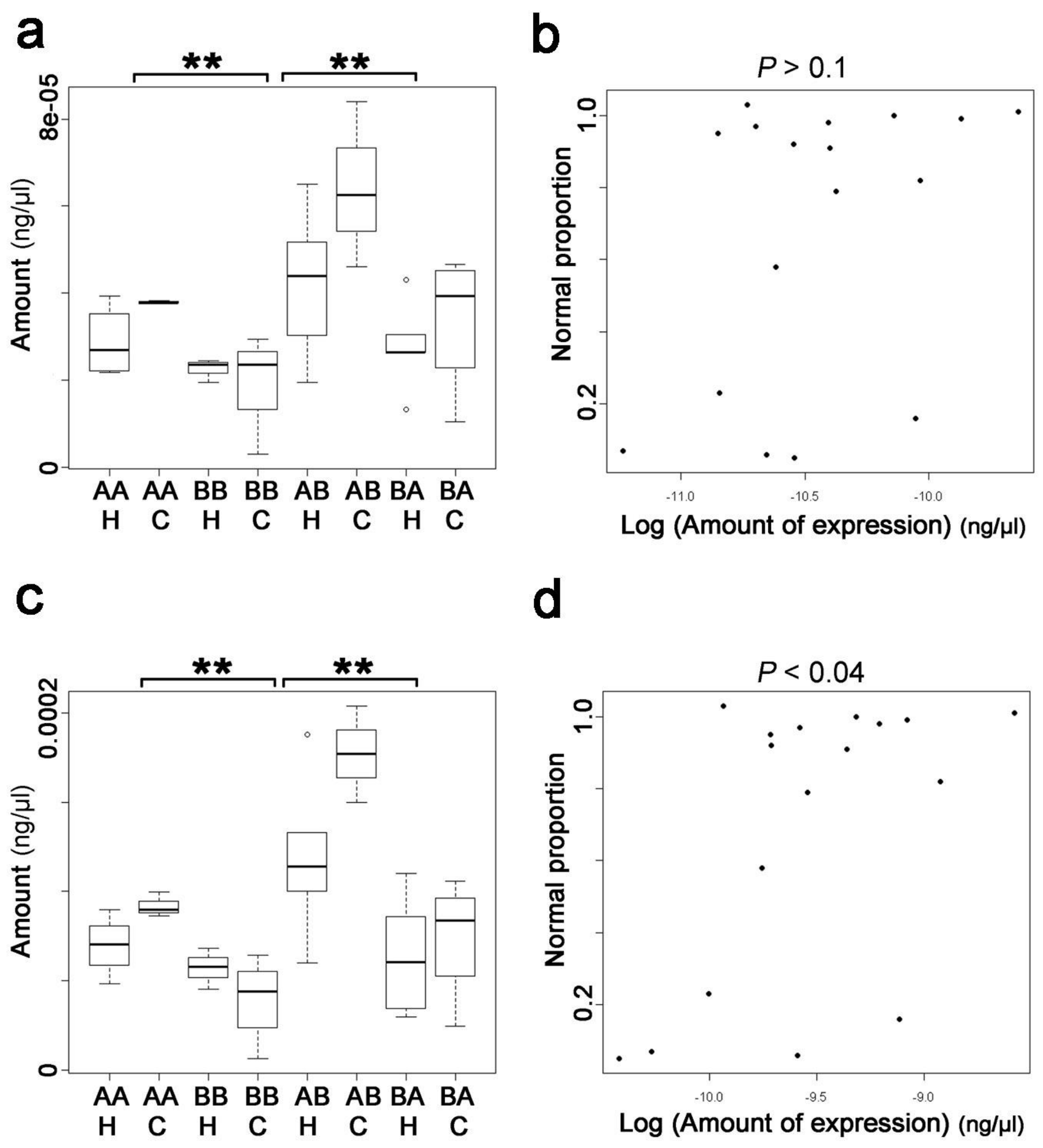

Supplementary Fig. 8. Comparison of expression levels of *dnajc5*. a, b, show results from primer set 1, whereas **c**, **d** show primer set 2. **a, c,** Quantification of *dnajc5* expression levels by qPCR in control (C) or heat shocked (H: 27ºC for 1h at neurula stage as in Fig. 1a) embryos. Heat shock did not significantly affect the expression of *dnajc5* ($P > 0.2$), and type A (AA) was significantly higher than in type B (BB) in the expression levels only under control condition ($P = 0.0029$). Reciprocal hybridization (AB/BA) did not show this was linked to the maternal genotype ($P = 0.0023$). Significances are shown by asterisks: $**P < 0.01$. **b, d,** Correlation between the amount of *dnajc5* transcripts and Normal proportion after heat shock in individual crosses. Analysis of Variance (ANOVA) did not supports linear correlation of the amount of *dnajc5* transcripts and normal proportion ($P = 0.1197$ for primer set 1 and $P = 0.03274$ for primer set 2).

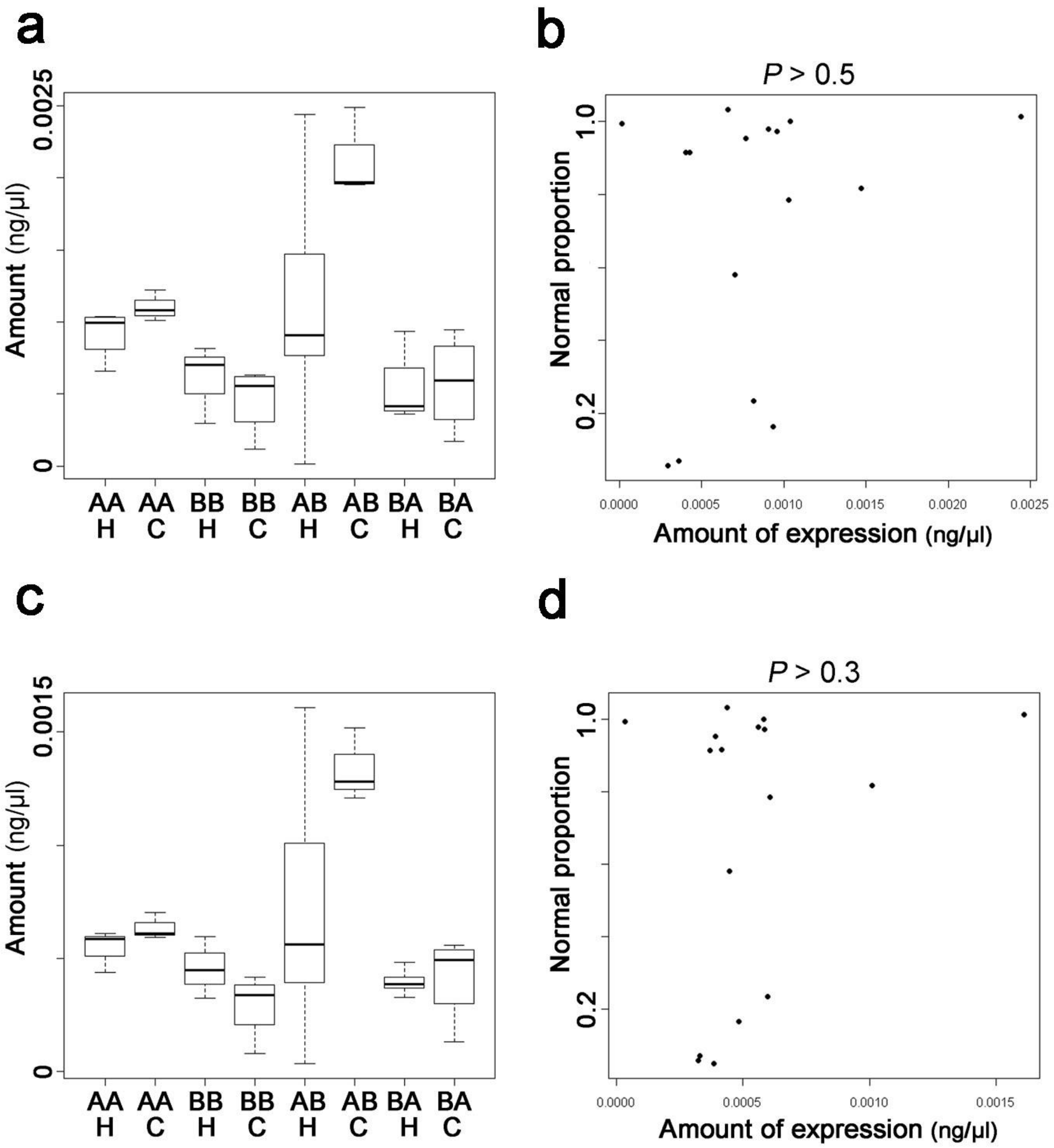

Supplementary Fig. 9. **Comparison of expression levels of *dnajb9*. a, b,** show results from primer set 1, whereas **c**, **d** show primer set 2. **a, c,** Quantification of *dnajb9* expression levels by qPCR in control (C) or heat shocked (H: 27ºC for 1h at neurula stage as in Fig. 1a) embryos. Heat shock did not significantly affect the expression of *dnajb9* ($P < 0.4$), and expression in type A (AA) did not show significant difference from expression in type B (BB). However, reciprocal hybridization (AB/BA) suggests this was linked to the maternal genotype ($P = 0.0061$). **b, d,** Correlation between the amount of *dnajb9* transcripts and Normal proportion after heat shock in individual crosses. Analysis of Variance (ANOVA) did not support linear correlation of the amount of *dnajb9* transcripts and normal proportion ($P = 0.554$ for primer set 1, and $P = 0.335$ for primer set 2).

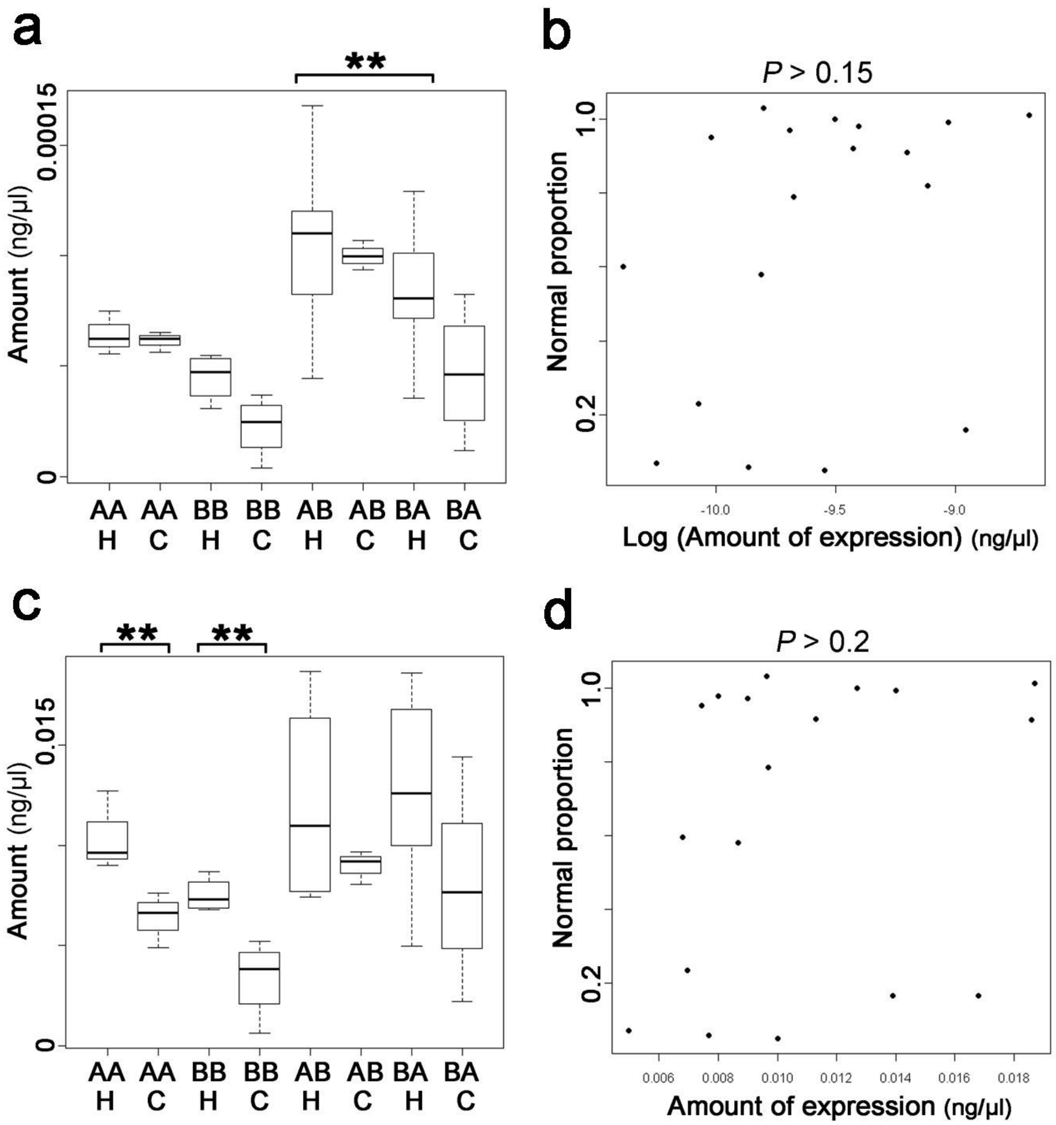

Supplementary Fig. 10. Comparison of expression levels of *hspa9b* and *hspa8*. a, b, show results from *hspa9b*, whereas **c**, **d** show *hspa8*. **a, c,** Quantification of each gene expression levels by qPCR in control (C) or heat shocked (H: 27ºC for 1h at neurula stage as in Fig. 1a) embryos. Expression in type A (AA) did not show significant difference from expression in type B (BB) in both genes (for *hspa9b* P = 0.0366, for *hspa8* P = 0.0882). Heat shock significantly affected the expression of *hspa8* (P = 0.0021) but not in *hspa9b* (P = 0.048). Reciprocal hybridization (AB/BA) marginally supported this was linked to the maternal genotype in *hspa9b* (P = 0.0066) but not for *hspa8* (P = 0.8298). Significances are shown by asterisks: **P <0.01. **b, d,** Correlation between the amount of each gene transcripts and Normal proportion after heat shock in individual crosses. Analysis of Variance (ANOVA) did not support linear correlation of the amount of transcripts and normal proportion in neither of these genes (P=0.1575 for *hspa9b* and P=0.2384 for *hspa8*).

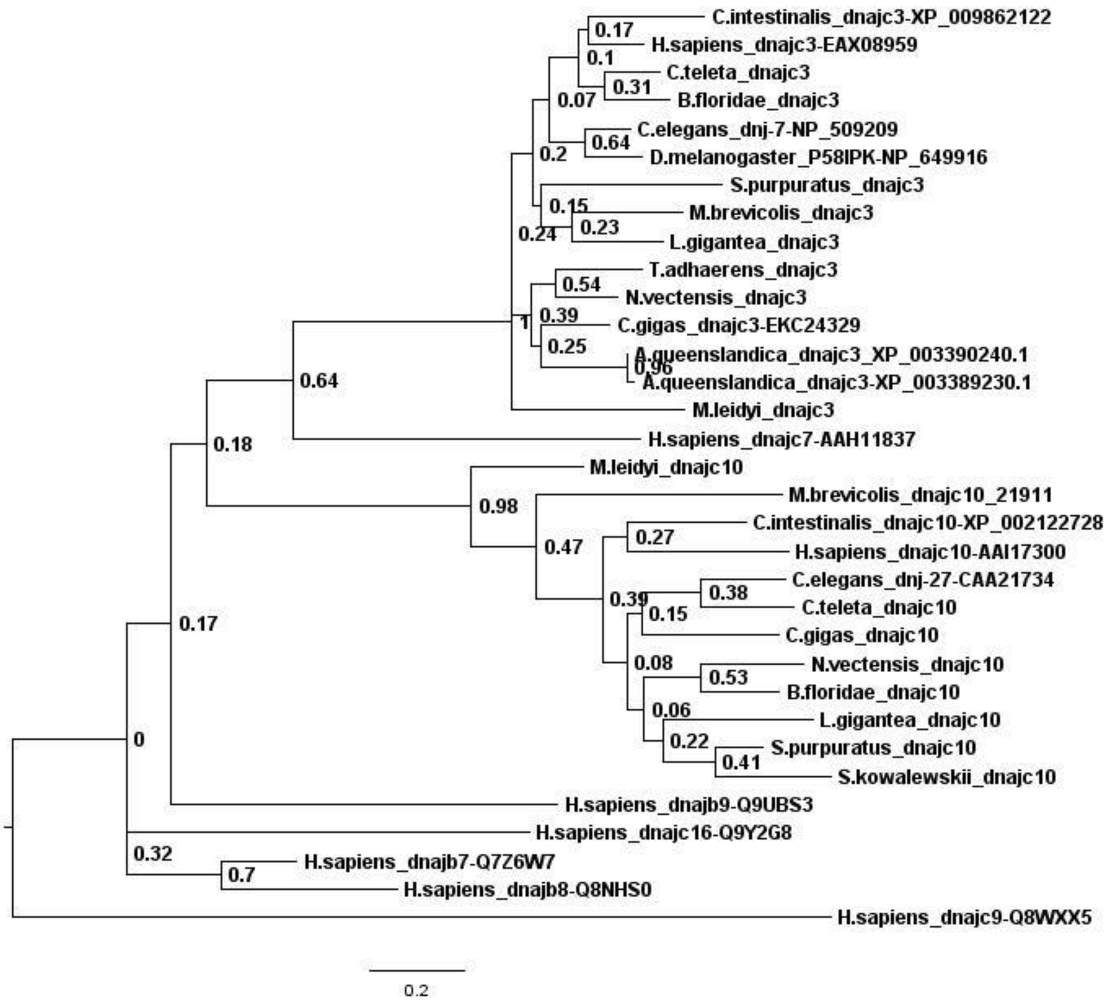

Supplementary Fig. 11. Maximum Likelihood analysis of *dnajc3* and *dnajc10* genes. Species used for the tree are as follows: *Drosophila melanogaster*, *Caenorhabditis elegans*, *Homo sapiens*, *Ciona intestinalis*, *Branchiostoma floridae* (amphioxus), *Strongylocentrotus purpuratus* (sea urchin), *Saccoglossus kowalevskii* (acorn worm), *Lottia gigantea* (mollusc), *Crassostrea gigas* (mollusc), *Capitella teleta* (annelid), *Nematostella vectensis* (anemone), *Mnemiopsis leidyi* (ctenophore), *Amphimedon queenslandica* (sponge), *Monosiga brevicolis* (choanoflagellate). Accession numbers are given in the figure for outgroup sequences and sequences from model species, for other sequence sources please refer to Supplementary Table S7.
